# Maternal region of origin and Small for gestational age: a cross-sectional analysis of Victorian perinatal data

**DOI:** 10.1186/s12884-021-03864-9

**Published:** 2021-05-29

**Authors:** Sarah Grundy, Patricia Lee, Kirsten Small, Faruk Ahmed

**Affiliations:** 1 School of Medicine, Griffith University, Gold Coast, QLD Australia; 2 School of Nursing and Midwifery, Griffith University, QLD Gold Coast, Australia; 3 Transforming Maternity Care Collaborative, Griffith University, Gold Coast, QLD Australia

**Keywords:** Birthweight, Maternity, Migration, Perinatal health, Pregnancy, Region of origin, Small for gestational age

## Abstract

**Background:**

Being born small for gestational age is a strong predictor of the short- and long-term health of the neonate, child, and adult. Variation in the rates of small for gestational age have been identified across population groups in high income countries, including Australia. Understanding the factors contributing to this variation may assist clinicians to reduce the morbidity and mortality associated with being born small. Victoria, in addition to New South Wales, accounts for the largest proportion of net overseas migration and births in Australia. The aim of this research was to analyse how migration was associated with small for gestational age in Victoria.

**Methods:**

This was a cross sectional population health study of singleton births in Victoria from 2009 to 2018 (*n* = 708,475). The prevalence of being born small for gestational age (SGA; <10th centile) was determined for maternal region of origin groups. Multivariate logistic regression analysis was used to analyse the association between maternal region of origin and SGA.

**Results:**

Maternal region of origin was an independent risk factor for SGA in Victoria (*p* < .001), with a prevalence of SGA for migrant women of 11.3% (*n* = 27,815) and 7.3% for Australian born women (*n* = 33,749). Women from the Americas (aOR1.24, 95%CI:1.14 to 1.36), North Africa, North East Africa, and the Middle East (aOR1.57, 95%CI:1.52 to 1.63); Southern Central Asia (aOR2.58, 95%CI:2.50 to 2.66); South East Asia (aOR2.02, 95%CI: 1.95 to 2.01); and sub-Saharan Africa (aOR1.80, 95%CI:1.69 to 1.92) were more likely to birth an SGA child in comparison to women born in Australia.

**Conclusions:**

Victorian woman’s region of origin was an independent risk factor for SGA. Variation in the rates of SGA between maternal regions of origin suggests additional factors such as a woman’s pre-migration exposures, the context of the migration journey, settlement conditions and social environment post migration might impact the potential for SGA. These findings highlight the importance of intergenerational improvements to the wellbeing of migrant women and their children. Further research to identify modifiable elements that contribute to birthweight differences across population groups would help enable appropriate healthcare responses aimed at reducing the rate of being SGA.

## Background

Improving the wellbeing of women and children is essential if we are to achieve progress on the Sustainable Development Goals, reduce inequality and create a more inclusive future for all [1]. Every person’s life potential is shaped during the critical periods of growth and human development associated with conception, pregnancy, and birth. Growing small for gestational age (SGA; < 10th centile) in utero more than doubles the risk of stillbirth [2], increases the child’s risk for neonatal death [3], postnatal growth stunting [4], and reduces learning potential in comparison to a child born appropriate for gestational age (AGA) [5, 6]. In adulthood, individuals who were born SGA are predisposed to chronic health problems, resulting in a decreased earning capacity [7], reduced productivity, and increased economic costs for the broader population [8].

Reducing preventable perinatal death by achieving a reduction to 10% in the prevalence of SGA by 2035 is a target of the Every Newborn Action Plan [9] endorsed by the World Health Organization. Children born in low- and middle-income countries are more likely to be SGA, and there are also differences between population groups in high income countries [10]. In Australia, 11.9% of migrant children were born SGA for the year 2017 compared to 9.7% of those to Australian born women [11], raising questions of what factors are driving differences in the prevalence of SGA across population groups.

The causes of growth restriction during the antenatal period are multifactorial, ranging from fetal malformations, infections, placental and umbilical cord abnormalities; to maternal factors, such as maternal preeclampsia [12], diabetes [13], and anaemia [14]. In the absence of intrauterine pathology, SGA is associated with the intersection of a woman’s health and health behaviours in the context she inhabits [15–17]. Being a younger woman [18], not in a relationship [19, 20], or being the woman’s first baby [21] are associated with higher rates of SGA; as are smoking [22], being underweight, or having low gestational weight gain [23, 24]. In contrast, being overweight [13] and high gestational weight gain decreased the risk of SGA [25]. Gestational weight gain is primarily influenced by food security [26]. Food security is influenced by a complex intersection of factors such as socioeconomic status, geographical access to nutritional food, and health literacy [27]. Certain cultural factors are also associated with SGA through the influence of body image [28], food taboos, and dietary misconceptions [29, 30] on gestational weight gain.

Globally, environmental factors such as socioeconomic disadvantage [31, 32], natural disasters, famine, and conflict also increase the risk of SGA via pathways of reduced access to clean water, sanitation, food security, and health care [33–35]. High income countries have historically been more resilient to these factors, however, the increasing frequency of natural hazards such as bushfires, flooding and pandemics are contributing to social instability for all regions [36]. Variation in the risk of SGA in high income countries, including Australia, has been associated with a woman’s socioeconomic status [31, 32], via factors such as education [37], income [38], food deserts [27] and living conditions [19] that influence a woman’s access to resources in her social context. Socioeconomic status and the risk for SGA has been found to be influenced by a woman’s racial classification [39] or ethnic group [40], as race modifies exposure to racially determined disadvantage and systemic racism [41]. Meta-analyses of the factors associated with SGA for migrant women in high income countries have confirmed that a woman’s region of origin and migration status [42] increased her risk of SGA via pathways of access and barriers to social resources.

Australia was founded in the context of migration. The history of colonisation of First Nations country has resulted in Australia being home to people from over 270 diverse ancestry groups [43]. By 2018, close to 40% of women birthing in Australia were born overseas, with the largest proportion of women arriving from India and China [44]. Victoria and New South Wales account for the largest proportion of net overseas migration and births in Australia [43, 45]. Maternal region of birth was found to be an independent risk factor for stillbirth and this risk increased 2.3-fold when SGA was diagnosed and 4.3-fold when SGA was not recognised in utero [46]. Previous migrant health research in Victoria has identified poorer perinatal health outcomes for some migrant population groups [47]. Population group differences in the prevalence of SGA have been identified for migrant women in high income countries. However, possible differences in rates of SGA across population groups in Victoria remains unknown. This lack of knowledge is potentially contributing to a higher risk of stillbirth for women from certain regions of origin. Therefore, the aim of this study was to determine how a woman’s position as a migrant is associated with the prevalence of being SGA at birth in Victoria.

## Methods

### Study design and population

A cross-sectional study of routinely collected population data on all singleton births in Victoria between January 2009 to December 2018 was undertaken. The quality of the Victorian Perinatal Data Collection (VPDC) is regularly audited for accuracy which supports the validity of the findings in this research study [48]. Data were available for the total population of women (*n* = 708, 475), providing a sufficiently large study population for analysis across subgroups. Exclusion criteria were: fetal deaths, stillbirths, congenital abnormalities, births at gestations of less than 20 or greater than 43 weeks, and unknown neonatal sex, gestation, or birthweight. Multiple births were also excluded as they are more likely to be confounded by prematurity and maternal pregnancy conditions. After data cleaning, the sample captured 98.9% of women birthing a singleton baby in Victoria during the time period.

Low risk ethics approval was granted in December 2019 for secondary analysis of routinely collected perinatal data. All research methods were performed in accordance with the relevant guidelines and regulations for the analyses of secondary population data. The Consultative Council on Obstetric and Paediatric Mortality and Morbidity (CCOPMM) provided formal access to the deidentified data according to regulation 10 of the Public Health and Wellbeing Regulations 2009, ensuring respect for the privacy and cultural sensitivities of the women included in the study. Ethical review applied the Victorian Health Records Act Statutory Guidelines for research and due to the deidentified nature of the data a waiver for consent was approved. The research data were stored according to quality procedures for the storage of research data, including data encryption for transfer and storage.

### Measurement of key variables

#### Small for gestational age

Small for gestational age was the dependant variable, defined as a birthweight <10th centile, adjusted for sex and gestational age [49]. Birthweight centiles were determined using the Australian national birthweight percentiles to define the point of significance [50] and were added by the VPDC prior to release to the researchers. The population standard was chosen as it has been validated to be representative of the Australian population [50], is the standard used in Victoria for population health research [51], and does not assume that race or ethnicity generates genetic differences in birthweight [52].

#### Maternal region of origin

The independent variable, maternal region of origin, was a composite categorical variable provided by the Victorian Agency for Health Information (VAHI) and grouped according to the United Nations M49 Standard geoscheme [53]. Country of birth is a self-reported indicator routinely collected during the provision of maternity care in Victoria [54]. In preparation for analysis, we consolidated maternal country of birth into two categories, non-migrant women born in Australia and migrant women who were born in a country other than Australia. The variable region of origin was further grouped according to the Australian Bureau of Statistics Classification of Countries [55] into major regional groupings: Americas; Australia; Europe; North Africa, North East Africa and Middle East; Oceania and Antarctica; Southern Central Asia; South East Asia; and sub-Sharan Africa.

#### Potential confounding variables

Socioeconomic Indexes for Areas (SEIFA) was used as the measure for socioeconomic status. SEIFA is a composite measure that includes a range of variables including the level of relative disadvantage/advantage in an area, employment status, household income, level of education, disability, single parenthood, and rental or mortgage status [56]. The SEIFA quintiles were consolidated from 5 levels into SEIFA advantaged (quintiles 4 and 5) average (quintile 3) and disadvantaged (quintiles 1 and 2) for analysis. Additional variables controlled for were provided to the researchers in the data set and detailed definitions can be found in the Victorian Perinatal Data Collection (VPDC) definitions manual [54]. Variables included maternal age in years (less than 20 years, 20 to 35 years, over 35 years); parity as the total number of previous pregnancies (multiparous and primiparous); relationship status described a woman’s existing relationship status, (in a relationship or not in a relationship); body mass index (BMI < 18.5 underweight, BMI 18.5 to 25 average weight, BMI > 25 overweight or obese); smoking status before and after 20 weeks gestation (smoking or non-smoking), gestational age at first pregnancy visit (< 12 weeks, 12 to 24 weeks, > 24 weeks/no care). Maternal medical conditions and pregnancy complications (categorised yes - disorder present or no - disorder absent) included, diabetes, gestational diabetes; pre-existing hypertension and pregnancy induced hypertension (blood pressure systolic ≥140 mmHg and/or diastolic ≥90 mmHg). Pre-eclampsia, HELLP syndrome (haemolysis, elevated liver enzymes, low platelet count) were defined by the SOMANZ guideline for management of hypertensive disorders of pregnancy [57] and suspected fetal growth restriction (clinically determined slow/static growth <10th centile) [58].

### Statistical analysis

The statistical data editor software program Statistical Product and Services Solution version 26 (SPSS V.26) was used for analyses. Data were checked using frequencies, errors, and outliers in preparation for analyses. Of the total study population, 704 cases (0.01%) were missing birthweight and these cases were deleted. Bivariate analyses using the Chi-square test for independence between the dependant variable SGA and the independent variable were performed, including examining the potential confounding of mediating variables identified in the literature review. Statistically significant unadjusted associations were identified in preparation for binary logistic regression analysis. Hierarchical logistic regression models were built to measure the independent association [presented as adjusted odds ratio (aOR) with 95% confidence intervals (95%CI)] between SGA and maternal region of origin, whilst controlling for effects of the confounding variables defined in the previous section. Statistical significance was set at *p* value < 0.05.

## Results

Between 2009 and 2018 there were 708,475 women who birthed a singleton baby in Victoria. The demographics of the study population are presented in Table 1. Most women were born in Australia (67%; *n* = 461,903). The remaining women were migrants from the Americas (1.4% *n* = 9616); Europe (7.9% *n* = 34,533); Northeast Africa, North Africa, Middle East (7.9% *n* = 55,737); Oceania, Antarctica (2.8% *n* = 20,046); Southern Central Asia (9.4% *n* = 66,883); South East Asia (6.3% *n* = 44,943); and sub-Saharan Africa (2.1% *n* = 14,814). A smaller proportion of migrant women than Australian born women were not in a relationship (6.7% vs 14.6% respectively); under the age of 20 years (0.8% vs 2.5%); and living in a more advantaged neighbourhood (36.9% vs 41.6%). Migrant women were also more likely than Australian born women to be birthing their first baby (45.3% vs 43% respectively); develop more gestational diabetes managed with diet (9.5% vs 4.0%) and insulin (6.1% vs 2.8%); and experience more suspected fetal growth restriction (5.8% vs 4.3%). The majority of women in the study population were non-smokers. Those who did smoke were more likely to be Australian born women rather than migrant women, both before 20 weeks gestation (13.4% vs 3.8%); and after 20 weeks gestation (8.5% vs 1.9%). These findings were statistically significant at *p* <  0.001.

**Table 1:** Unadjusted characteristics of the study population (*n* = 708,475) stratified maternal region of origin

Region of Origin	Australia	Americas	Europe	North East, North Africa, Middle East	Oceania, Antarctica	South East Asia	Southern, Central Asia	Sub-Saharan Africa	
	***n*** = 461,903 (65.2%)	***n*** = 9616 (1.4%)	***n*** = 34,533 (7.9%)	***n*** = 55,737 (7.9%)	***n*** = 20,046 (2.8%)	***n*** = 44,943 (6.3%)	***n*** = 66,883 (9.4%)	***n*** = 14,814 (2.1%)	
Demographics	N (%)	N (%)	N (%)	N (%)	N (%)	N (%)	N (%)	N (%)	***p*** value
**Birthweight**									
< 10th Centile	33,749 (7.3)	769 (8.0)	2397 (6.9)	5563 (10.0)	1399 (7.0)	5485 (12.1)	10,705 (16.0)	1524 (10.3)	< 0.001
> 10th Centile	428,154 (92.7)	8847 (92.0)	32,136 (93.1)	50,174 (90.0)	18,647 (93.0)	39,485 (87.9)	56,178 (84.0)	13,290 (89.7)	
**Sex - Baby**									
Male	235,529 (51.0)	4821 (50.1)	17,646 (51.1)	28,648 (51.4)	10,264 (51.2)	23,044 (51.3)	34,307 (51.3)	7504 (50.7)	= 0.179
Female	226,374 (49.0)	4795 (49.9)	16,887 (48.9)	27,089 (48.6)	9782 (48.8)	21,899 (48.7)	32,576 (48.7)	7310 (49.3)	
**Gestational Age Grouped**									
Preterm < 37 weeks	63,312 (13.7)	1186 (12.3)	4065 (11.8)	6334 (11.4)	2686 (13.4)	6665 (14.8)	10,117 (15.1)	1849 (12.5)	< 0.001
Term ≥37 weeks	398,591 (86.3)	8430 (87.7)	30,468 (88.2)	49,403 (88.6)	17,360 (86.6)	38,278 (85.2)	56,766 (84.9)	12,965 (87.5)	
**Relationship Status**									
In a Relationship	388,233 (85.4)	8988 (94.4)	32,154 (94.0)	51,882 (93.7)	16,440 (82.9)	40,559 (90.9)	65,873 (98.8)	12,639 (85.9)	< 0.001
Not in a relationship	66,453 (14.6)	538 (5.6)	2039 (6.0)	3492 (6.3)	3400 (17.1)	4048 (9.1)	810 (1.2)	2077 (14.1)	
**Maternal Age Grouped**									
< 20	11,703 (2.5)	32 (0.3)	124 (0.4)	503 (0.9)	559 (2.8)	285 (0.6)	156 (0.2)	192 (1.3)	< 0.001
20–35	358,684 (77.7)	6822 (71.0)	24,206 (70.1)	44,953 (80.7)	15,223 (76.0)	34,707 (77.2)	60,138 (89.9)	11,368 (76.7)	
35+	91,311 (19.8)	2757 (28.7)	10,187 (29.5)	10,274 (18.4)	4257 (21.2)	9945 (22.1)	6579 (9.8)	3252 (22.0)	
**Parity**									
Multiparous	263,087 (57.0)	5057 (52.6)	18,783 (54.4)	30,998 (55.6)	12,692 (63.3)	25,237 (56.2)	32,205 (48.2)	9767 (66.0)	< 0.001
Nulliparous	198,771 (43.0)	4557 (47.4)	15,745 (45.6)	24,734 (44.4)	7349 (36.7)	19,701 (43.8)	34,670 (51.8)	5041 (34.0)	
**Maternal SEIFA**									
Advantage	179,694 (41.6)	4863 (53.4)	17,377 (53.1)	20,896 (39.3)	6616 (34.8)	13,599 (31.6)	18,465 (29.2)	4774 (33.9)	< 0.001
Average	89,356 (20.7)	1703 (18.7)	6218 (19.0)	9766 (18.3)	3741 (19.7)	6938 (16.1)	13,194 (20.8)	2115 (15.0)	
Disadvantage	162,815 (37.7)	2541 (27.9)	9144 (27.9)	22,571 (42.4)	8679 (45.6)	22,511 (52.3)	31,651 (50.0)	7189 (51.1)	
**Maternal Health and Health Behaviours**									
**Body Mass Index**									
< 18.5	12,653 (2.9)	259 (2.9)	905 (2.8)	1663 (3.2)	520 (2.8)	1434 (3.4)	2242 (3.6)	416 (3.1)	< 0.001
18.5–24.99	215,510 (50.2)	4521 (51.1)	15,999 (50.2)	26,570 (51.6)	9257 (49.5)	21,814 (52.3)	32,425 (52.1)	6891 (51.0)	
> 25	201,141 (46.9)	4071 (46.0)	14,972 (47.0)	23,278 (45.2)	8919 (47.7)	18,435 (44.2)	27,541 (44.3)	6211 (45.9)	
**Smoking before 20wks**									
Smoker	60,786 (13.4)	366 (3.9)	2469 (7.2)	1490 (2.7)	3296 (16.8)	968 (2.2)	285 (0.4)	445 (3.0)	< 0.001
Non-Smoker	393,058 (86.6)	9136 (96.1)	31,648 (92.8)	53,622 (97.3)	16,288 (83.2)	43,615 (97.8)	66,384 (99.6)	14,251 (97.0)	
**Smoking after 20wks**									
Smoker	32,872 (8.5)	114 (1.3)	951 (3.2)	613 (1.2)	1873 (10.8)	323 (0.8)	77 (0.1)	173 (1.3)	< 0.001
Non-Smoker	352,354 (91.5)	8413 (98.7)	28,943 (96.8)	50,486 (98.8)	15,421 (89.2)	40,277 (99.2)	61,892 (99.9)	13,060 (98.7)	
**Gestational Age 1st visit**									
Before 11 weeks	222,760 (48.8)	4391 (46.2)	15,280 (44.7)	21,824 (39.5)	6328 (32.0)	16,386 (36.8)	22,453 (33.8)	4786 (32.7)	< 0.001
12 to 23 weeks	202,110 (44.2)	4471 (47.0)	16,817 (49.2)	28,276 (51.2)	10,039 (50.7)	24,050 (54.0)	38,399 (57.8)	8210 (56.2)	
After 23 weeks/no care	31,976 (7.0)	644 (6.8)	2098 (6.1)	5139 (9.3)	3427 (17.3)	4113 (9.2)	5621 (8.5)	1691 (11.1)	
**Maternal Medical and Pregnancy Complications**									
**Type 1 Diabetes**									
Yes	1863 (0.4)	18 (0.2)	85 (0.2)	90 (0.2)	82 (0.4)	97 (0.2)	303 (0.5)	51 (0.3)	< 0.001
No	460,040 (99.6)	9598 (99.8)	34,448 (99.8)	55,647 (99.8)	19,964 (99.6)	44,846 (99.8)	66,580 (99.5)	14,763 (99.7)	
**Type 2 Diabetes**									
Yes	717 (0.2)	21 (0.2)	38 (0.1)	150 (0.3)	103 (0.5)	162 (0.4)	386 (0.6)	88 (0.6)	< 0.001
No	461,186 (99.8)	9595 (99.8)	34,495 (99.9)	55,587 (99.7)	19,943 (99.5)	44,781 (99.6)	66,497 (99.4)	14,726 (99.4)	
**Pre-existing Hypertension**									
Yes	6039 (1.3)	117 (1.2)	333 (1.0)	344 (0.6)	256 (1.3)	449 (1.0)	539 (0.8)	174 (1.2)	< 0.001
No	455,864 (98.7)	9499 (98.8)	34,200 (99.0)	55,393 (99.4)	19,790 (98.7)	44,494 (99.0)	66,344 (99.2)	14,640 (98.8)	
**Gestational Diabetes -Diet**									
Yes	18,506 (4.0)	467 (4.9)	1668 (4.8)	5379 (9.7)	1191 (5.9)	5283 (11.8)	8399 (12.6)	1046 (7.1)	< 0.001
No	443,397 (96.0)	9149 (95.1)	32,865 (95.2)	50,358 (90.3)	18,855 (94.1)	39,660 (88.2)	58,484 (87.4)	13,768 (92.9)	
**Gestational Diabetes - Insulin**									
Yes	12,769 (2.8)	315 (3.3)	974 (2.8)	2835 (5.1)	889 (4.4)	2503 (5.6)	6663 (10.0)	740 (5.0)	< 0.001
No	449,134 (97.2)	9301 (96.7)	33,559 (97.2)	52,902 (94.9)	19,157 (95.6)	42,440 (94.4)	60,220 (90.0)	14,074 (95.0)	
**Pre-Eclampsia**									
Yes	10,332 (2.2)	144 (1.5)	582 (1.7)	590 (1.1)	510 (2.5)	697 (1.6)	1205 (1.8)	309 (2.1)	< 0.001
No	451,571 (97.8)	9472 (98.5)	33,951 (98.3)	55,146 (98.9)	19,536 (97.5)	44,246 (98.4)	65,678 (98.2)	14,505 (97.9)	
**HELLP Syndrome**									
Yes	732 (0.2)	13 (0.1)	56 (0.2)	58 (0.1)	37 (0.2)	59 (0.1)	108 (0.2)	47 (0.3)	< 0.001
No	461,171 (99.8)	9603 (99.9)	34,477 (99.8)	55,679 (99.9)	20,009 (99.8)	44,844 (99.9)	66,775 (99.8)	14,767 (99.7)	
**Suspected Fetal Growth Restriction**									
Yes	19,766 (4.3)	370 (3.8)	1276 (3.7)	3156 (5.7)	784 (3.9)	2564 (5.7)	5454 (8.2)	681 (4.6)	< 0.001
No	442,137 (95.7)	9246 (96.2)	33,257 (96.3)	52,581 (94.3)	19,262 (96.1)	42,397 (94.3)	61,429 (91.8)	14,133 (95.4)	

1. *N (%)* number and percentage. 2. *BMI* body mass index. 3. *Suspected FGR* suspected fetal growth restriction. 4. *HELLP* haemolysis, elevated liver enzymes and low platelet count. 5. *SEIFA* socioeconomic indexes for areas

### Maternal region of origin and SGA

There was a total of 61,564 SGA neonates in the study population, resulting in an overall SGA rate of 8.7%. A higher proportion of SGA was identified for migrant women at 11.3% (*n* = 27,815) compared to 7.3% (*n* = 33,749) for Australian born women. Bivariate analysis identified statistically significant variation in the distribution of SGA across maternal regions of origin (*p* <  0.001). The proportion of SGA for migrant women from Southern Central Asia 16.0% (*n* = 10,705); sub-Saharan Africa 14% (*n* = 17,687); South East Asia 12.1% (*n* = 5458); North East, North Africa, and the Middle East 10% (*n* = 5563); was higher than for women from Australia 7.3% (n = 33,749); the Americas 8.0% (*n* = 769); Oceania, Antarctic 7.0% (*n* = 1399) and Europe 6.9% (*n* = 2397). After controlling for confounding factors, regression analysis identified migrant women were 1.76 times more likely to birth an SGA baby than women born in Australia (95%CI:1.73 to 1.80, *p* < .001), (see Table 2.).

**Table 2:** The association between known risk factors (including Migration Status) and SGA

Variable		SGA aOR (95%CI)
**Marital Status**	In a Relationship ^r^	1
	Not in a Relationship	1.08 (1.04 to 1.11) ***
**Age Group (years)**	< 20	0.90 (0.84 to 0.97) **
	20 to 35 ^r^	1
	35+	1.07 (1.04 to 1.10) ***
**Parity**	Primiparous	2.01 (1.97 to 2.06) ***
	Multiparous ^r^	1
**BMI**	Underweight	1.61 (1.54 to 1.69) ***
	Healthy Weight ^r^	1
	Overweight	0.77 (0.76 to 0.79) ***
**Smoking before 20wks**	Yes	1.12 (1.04 to 1.18) **
	No ^r^	1
**Smoking after 20wks.**	Yes	2.25 (2.10 to 2.41) ***
	No ^r^	1
**Gestational Age at 1st Visit**	Before 11 weeks ^r^	1
	12 to 23 weeks	1.09 (1.07 to 1.12) ***
	24 weeks /no care	1.25 (1.21 to 1.30) ***
**Type 1 Diabetes**	Yes	0.35 (0.27 to 0.45) ***
	No ^r^	1
**Type 2 Diabetes**	Yes	0.74 (0.59 to 0.93) *
	No ^r^	1
**Pre-existing Hypertension**	Yes	1.27 (1.16 to 1.38) ***
	No ^r^	1
**Gestational Diabetes - Diet**	Yes	1.04 (1.00 to 1.08) *
	No ^r^	1
**Gestational Diabetes - Insulin**	Yes	0.83 (0.78 to 0.87) ***
	No ^r^	1
**Preeclampsia**	Yes	1.45 (1.37 to 1.54) ***
	No ^r^	1
**HELLP Syndrome**	Yes	1.72 (1.42 to 2.08) ***
	No ^r^	1
**Suspected FGR**	Yes	10.47 (10.19 to 10.76) ***
	No ^r^	1
**Migration Status**	Australia Born Women ^r^	1
	Migrant Women	1.76 (1.73 to 1.80) ***

1. Birthweight Adjusted for Sex and Gestational Age. 2. *r* reference. 3. ***p < 0.001, ** *p* < 0.01, *p < 0.05

### The independent association between SGA and maternal region of origin

Binary logistical regression modelling was performed to identify the differences in SGA between maternal regions of origin groups (see Table 3). Prior to model building, multicollinearity was measured by variance inflation factors (VIF) and tolerance. For all predictor variables the tolerance was > 0.01 and VIF > 1 and < 3.5. Regression analysis identified a statistically significant independent association between SGA and maternal regions of origin. In comparison to Australian born women, migrant women from the Americas were 1.24 times more likely to birth an SGA child (95%CI: 1.14 to 1.36), North Africa, North East Africa, and the Middle East OR1.57 (95%CI:1.52 to 1.63), sub-Saharan Africa OR1.80 (95%CI:1.69 to 1.92), South East Asia OR 2.02 (95%CI:1.95 to 2.01) and Southern Central Asia OR 2.58 (95%CI:2.50 to 2.66).

**Table 3:** Adjusted odds ratio (95%CI) the association between SGA and maternal region of origin

Maternal Region of Origin	SGA aOR (95%CI)
Australia Born Women ^r^	1
Americas	1.24 (1.14 to 1.36) ***
North Africa, North East Africa, Middles East	1.57 (1.52 to 1.63) ***
Europe	1.03 (0.98 to 1.09)
Oceania, Antarctica	0.97 (0.91 to 1.40)
Southern Central Asia	2.58 (2.50 to 2.66) ***
South East Asia	2.02 (1.95 to 2.01) ***
Sub-Saharan Africa	1.80 (1.69 to 1.92) ***

1. Birthweight Adjusted for Sex and Gestational Age. 2. *r* reference. 3. ***p < 0.001. * *p* < 0.05. 4. Confounding variables included, marital status, maternal age group, parity, BMI, height group, smoking before or after 20 weeks gestation, gestational age at first visit, type 1 diabetes pre-existing hypertension, gestational diabetes -insulin, pre-eclampsia, HELLP syndrome, suspected FGR

Socioeconomic status has previously been identified as a strong influencing factor in the association with SGA and it is strongly associated with maternal region of origin χ^2^ (df 14, *N* = 57,759) = 782.33. *p* < .001 (data not shown). Therefore, further stratified regression analyses were undertaken to assess the independent effect of region of origin on SGA while removing its interrelationship with SEIFA. A series of modelling stratified by SEIFA advantaged, average, and disadvantaged was performed to confirm the relationships between SGA and region of origin (see.Table 4.). The independent association between maternal region of origin and SGA was statistically significant across all SEIFA groups except for women from Europe, Oceania and Antarctica or average and disadvantaged women from Americas. For migrant women in Victoria, the risk of SGA did not always follow a classic socioeconomic gradient of disadvantage, confirming maternal region of origin was a much stronger predictor of SGA than maternal socioeconomic status.

**Table 4:** The association between SGA and maternal region of origin stratified SEIFA advantage, average, disadvantaged

SGA Stratified by Maternal SEIFA, OR (95%CI)			
Maternal Region of Origin	SEIFA Advantaged	SEIFA Average	SEIFA Disadvantaged
Australia ^r^	1	1	1
Americas	1.46 (1.22 to 1.75) ***	1.00 (0.80 to 1.26)	1.22 (0.98 to 1.54)
North & North East Africa, Middle East	1.64 (1.51 to 1.78) ***	1.58 (1.45 to 1.72) ***	1.51 (1.38 to 1.65) ***
Europe	1.10 (0.98 to 1.22)	1.02 (0.90 to 1.15)	0.94 (0.83 to 1.08)
Oceania, Antarctica	1.04 (0.89 to 1.23)	0.97 (0.81 to 1.14)	0.95 (0.81 to 1.10)
South East Asia	2.06 (1.88 to 2.60) ***	2.10 (1.92 to 2.30) ***	1.84 (1.68 to 2.01) ***
Southern Central Asia	2.71 (2.52 to 2.91) ***	2.65 (2.48 to 2.83) ***	2.54 (2.38 to 2.72) ***
Sub–Saharan Africa	1.83 (1.75 to 1.91) ***	1.89 (1.60 to 2.23) ***	1.75 (1.48 to 2.06) ***

1. Birthweight Adjusted for Sex and Gestational Age. 2. *r* reference. 3. ***p < 0.001. 4. Confounding variables included, marital status, maternal age group, parity, BMI, height group, smoking before or after 20 weeks gestation, gestational age at first visit, type 1 diabetes pre-existing hypertension, gestational diabetes -insulin, pre-eclampsia, HELLP syndrome, suspected FGR

Being born SGA was also associated with several maternal characteristics, medical conditions, and pregnancy complications (see Table 2.). For the total study population, SGA was less likely for women under 20 years (aOR 0.90, 95%CI:0.72 to 0.85, *p* < .01), and more likely for women over 35 years (aOR 1.07, 95%CI:1.04 to 1.10, *p* < .001) when compared with women aged between 20 to 35 years. Women birthing their first baby were twice as likely (aOR 2.01, 95%CI:1.97 to 2.06, *p* < .001) to birth an SGA child than women birthing subsequent babies. Being underweight increased a woman’s odds of birthing an SGA baby (aOR 1.61, 95%CI:1.54 to 1.69, *p* < .001) and being overweight or obese decreased a woman’s odds of birthing an SGA baby (aOR 0.77, 95%CI:0.76 to 0.79, *p* < .001) when compared to women of a healthy weight.

Women unable to access care until after 24 weeks gestation or not at all were 25% more likely to birth an SGA child than women able to access maternity care before 12 weeks gestation (95%CI:1.21 to 1.30, *p* < .001). The risk of SGA increased in proportion to the severity of hypertensive disorders from 27% more likely with pre-existing hypertension, 45% more likely with pre-eclampsia, to 72% more likely with HELLP syndrome (*p* < .001). Type 1 diabetes (aOR 0.35, 95%CI:0.27 to 0.45, *p* < .001), type 2 diabetes (aOR 0.74, 95%CI:0.59 to 0.93, *p* < .05) and gestational diabetes treated with Insulin (aOR 0.83, 95%CI:0.78 to 0.87, *p* < .001), decreased the odds of SGA compared to women without these conditions. Women with suspected fetal growth restriction were over 10.47 times more likely to birth an SGA child than women not suspected of fetal growth restriction (95%CI:10.19 to 10.76, *p* < .001).

## Discussion

The prevalence of SGA in the population of Victorian women who gave birth between 2009 and 2018 was higher for migrant women than women born in Australia. When compared to Australian born women, the highest prevalence of SGA was for migrant women from the regions Southern Central Asia, South East Asia, and sub-Saharan Africa, followed by migrant women from North Africa, North East Africa, the Middle East, the Americas and was lowest for migrant women from the Europe, Oceania, and Antarctica. These findings were consistent after adjustment for potential confounding factors indicating a woman’s region of origin was a strong predictor of SGA in Victoria.

Women in our study population from regions with a higher proportion of low- and middle-income countries, such as Southern Central Asia, South East Asia and sub-Saharan Africa had a higher prevalence of SGA compared to women from high income regions such as Europe or Australia. These findings confirm a woman’s region of origin is associated with her risk for SGA. A potential explanation for this associated risk may be a woman’s preconception exposures to conditions that influence her reproductive health and therefore her potential to birth an SGA child. A woman’s context prior to migration shapes her wellbeing and sets the scene for her migration journey: growing up in a high-income country allows access to resources, such as universal education, food security and health care, that may not be available to all women in low- and middle-income countries.

The level of gender equality, conflict and stability of her environment influences a woman’s reason for migration: a voluntary migration for employment or education is a very different journey to one that is forced due to conflict or natural disaster [59]. In 2018, over 70 million people were escaping persecution and conflict, a forced migration leaving them exposed to human rights abuse, trauma, and human trafficking [60]. How a woman arrives in a new country determines her access to resources such as health care, employment, and freedom of movement, via complex visa systems and associated visa privileges or barriers [61]. Migration becomes a social determinant of SGA via pathways of the migration context [62], differential access to social resources during settlement [42, 63] and potential exposures to racially determined discrimination in a new country [41, 64, 65].

Previous research has identified the birthweights of migrant children increase over time to align with the birthweights of children born to women from the settlement country, irrespective of a woman’s geographical origin or ancestry [66]. Key factors in achieving this birthweight increase were comprehensive settlement policies that were responsive to the needs of migrant women [67]. This adjustment in birthweight after resettlement suggests the birthweight potential of migrant offspring is not fixed according to the woman’s ancestry, geographical origin, or preconception exposures. Rather, the context of migration, settlement conditions and the social environment post-migration also impact the potential for SGA [67].

Our research was not designed to measure the influence of pre- and post-migration conditions; however, we were able to identify additional factors to region of origin and SEIFA that were associated with SGA in Victoria. Consistent with other studies, we identified underweight women were more likely to birth an SGA child than women who were a healthy weight [13, 23, 24, 26, 68], confirming the importance of food security and appropriate gestational weight gain during pregnancy. As previously described, appropriate gestational weight gain is a modifiable factor influenced in part by access to social resources [13, 26]. In 2020, over 2 million migrants in Australia were on temporary student or work visas and ineligible to access social safety nets, the largest proportion of these being migrants from Asian regions [69, 70]. Migrant women from Southern Central Asia and South East Asian regions experienced the highest estimated risk of SGA in this study population.

We also identified women who were unable to access maternity care until after 24 weeks gestation or not at all were more likely to birth an SGA child when compared to women able to access care prior to 24 weeks gestation. Previous studies have found migrant Asian and African women in Victoria were more likely to present late for pregnancy care and to have poor or no care [71]. There are many structural barriers that create inequality in access to pregnancy care in high income countries [42, 72, 73]. The tiered system of public and private funded maternity care in Australia creates a financial barrier for migrant women on temporary visas. These women are ineligible for Medicare funded maternity care unless there is a reciprocal arrangement between Australia and their country of origin [69]. The mandated pre migration private health insurance associated with temporary student and work visas often results in no pregnancy cover or significant out of pocket the costs [74].

Migrant women have also reported experiences of systemic racism both in their everyday context and whilst accessing maternity care in Australia [75–77]. A review of non-English speaking migrant women’s experiences of maternity care in Victoria [78] identified no evidence of improvements over an 8-year period. Experiences of systemic racism in the context of factors such as geographical location, health literacy and staff cultural competency create barriers for migrant women to access maternity care [77]. Our findings of a decreased ability to access maternity care associated with an increased potential to birth an SGA child highlights the importance of addressing the barriers pregnant women experience to access culturally safe maternity care and social resources in Victoria.

### Strengths and limitations

The strength of this study is the large sample size which enabled analyses for the outcome of SGA across smaller population subgroups. Further, the quality of the VPDC has previously been validated for conservative analysis of associations which strengthens the generalisability of the findings to the population of Victoria. Data was predominantly complete on the key variables SGA and maternal region of origin; therefore, missing data was not a major problem for analysis. The study controlled for a number of confounding factors identified in previous research, which facilitated examination of independent associations. Some of the findings from our research regarding the influence of confounding factors and their association with SGA were similar to those from previous studies.

Our study design was unable to establish causal relationships between SGA and migrant status but does demonstrate an association between a woman’s region of origin and SGA. The accuracy of self-reported region of origin has not been established. However, the measure has been validated to be useful for initial analyses of health outcomes for migrant women in population data [48]. The concept of socioeconomic status are complex, and use of SEIFA as a broad measure of relative socioeconomic status prevents the identification of which elements of the SEIFA variable contribute to differences in health [56]. The data set used for analysis did not permit the identification of what elements of being a migrant woman, what countries in each region, or what elements of the SEIFA measure contributed to the risk of SGA.

In the present study we conducted regression analysis for odds of SGA irrespective of gestational age which may raise question of the influence of prematurity on the results as premature babies are more likely to be SGA and spontaneous prematurity is also influenced by a range of sociocultural factors. It is important to note that we did perform logistic regression analysis for the term >37wks cases to give insight into the potential influence of prematurity on the odds of SGA. Review of aOR’s for term neonates indicated minimal difference for aOR and 95%CI across regions of origin when compared to the analysis of aOR and 95%CI for all gestations combined. Therefore, we presented the odds ratio for SGA using the total SGA and gestational age was adjusted for in the analysis.

Prematurity is also a recognised limitation when using population growth standards [79]. In response, some authors would advocate for the use of a customised growth standard rather than a population growth standard to negate the confounding effect of prematurity [52]. However, customised growth standards cannot distinguish between the physiological or pathological characteristics of fetal growth [80] and thus may mask pathological growth restriction [79]. In addition, customising fetal growth standards assume a baby may be constitutionally small based on the mother’s geographical origins or ethnicity. Whilst there may always be babies born healthy and small, the INTERGROWTH-21st studies [17] have demonstrated improvements in fetal and neonatal wellbeing can be achieved across all population groups. When a woman’s health and nutrition needs were met in an environment conducive to wellbeing, only 3.5% of variation in fetal and newborn growth across population groups was due to differences in ancestry [16].

This study has addressed an important question regarding SGA that has direct clinical relevance. Understanding the different factors that lead to population group differences in being born SGA is essential if we are to address these and achieve an equitable and inclusive future for all women and children. A balanced approach is required to identify effective preventative strategies that do not contribute to inappropriate intervention in pregnancy for healthy small babies. To our knowledge this is the first study to measure the association between migration and SGA for the total population of women birthing in Victoria.

## Conclusions

A woman’s region or origin was found to be an independent factor associated with birthing an SGA child in Victoria, suggesting elements of migration and settlement contribute to significant differences in the risk associated with SGA. These findings indicate a call to action on both short- and long-term commitments to targeted initiatives is required. Ensuring access to maternity care is an essential first step to achieve intergenerational improvements in the wellbeing of migrant women and their children. Further research is required to determine which aspects of the migration and settlement experiences can be modified to reduce the risk of SGA and therefore avoid the long-term consequences that flow from this.

## Data Availability

The data that support findings of this study are under restrictions from the Victorian Perinatal Data Collection. The data were provided by the Victorian Agency for Health Information via their data request hub and are not publicly available. Due to the sensitive nature of public health records, access to the data is by formal application to the Consultative Council on Obstetric and Paediatric Mortality and Morbidity (CCOPMM).

## Abbreviations

AGA: Appropriate for gestational age
aOR: Adjusted Odds Ratio
BMI: Body mass index
CI: Confidence interval
EFW: Estimated fetal weight
FGR: Fetal growth restriction
HELLP: Haemolysis, elevated liver enzymes and low platelet count
INTERGROWTH-21ST: The international fetal and new-born growth consortium for the twenty-first century project
SEIFA: Socioeconomic indexes for areas
SGA: Small for gestational age
SOMANZ: Society of Obstetric Medicine of Australia and New Zealand
SPSS: Statistical product and services solution
VAHI: Victorian Agency for Health Information
VPDC: Victorian perinatal data collection

## References

[1] United Nations (2015). Transforming our world: the 2030 agenda for sustainable development.

[2] Flenady V, Wojcieszek A, Middleton P, Ellwood D, Erwich J, Coory M (2016). Stillbirths: recall to action in high-income countries. Lancet.

[3] Flamant C, Gascoin G (2013). Short-term outcome and small for gestational age newborn management. J Gynecol Obstet Biol Reprod.

[4] Lee A, Kozuki N, Cousens S, Stevens G, Blencowe H, Silveira M (2017). Estimates of burden and consequences of infants born small for gestational age in low- and middle-income countries with INTERGROWTH-21 st standard: Analysis of CHERG datasets. Br Med J (Online)..

[5] de Bie HMA, Oostrom KJ (2010). Delemarre-van de Waal HA. Brain development, intelligence and cognitive outcome in children born small for gestational age. Hormone Res Paediatr.

[6] Miller SL, Huppi PS, Mallard C (2016). The consequences of fetal growth restriction on brain structure and neurodevelopmental outcome. J Physiol.

[7] Australian Institute of Health and Welfare (2018). Australia's Health 2018.

[8] Risnes KR, Vatten LJ, Baker JL, Jameson K, Sovio U, Kajantie E, Osler M, Morley R, Jokela M, Painter RC, Sundh V, Jacobsen GW, Eriksson JG, Sørensen TIA, Bracken MB (2011). Birthweight and mortality in adulthood: a systematic review and meta-analysis. Int J Epidemiol.

[9] Lawn JE, Blencowe H, Oza S, You D, Lee ACC, Waiswa P, Lalli M, Bhutta Z, Barros AJD, Christian P, Mathers C, Cousens SN (2014). Every newborn: Progress, priorities, and potential beyond survival. Lancet.

[10] World Health Organization Global nutrition targets 2025: Low birth weight policy brief. Geneva: World Health Organization; 2014.

[11] Data tables: Australia's mothers and baby's 2017. AIHW Website. 2019. Available from: https://www.aihw.gov.au/reports/mothers-babies/australias-mothers-and-babies-2017-in-brief/data. [cited 20/12/2020]

[12] Burton GJ, Redman CW, Roberts JM, Moffett A (2019). Pre-eclampsia: pathophysiology and clinical implications. Br Med J.

[13] Denize KM, Acharya N, Prince SA, da Silva DF, Harvey ALJ, Ferraro ZM (2018). Addressing cultural, racial and ethnic discrepancies in guideline discordant gestational weight gain: a systematic review and meta-analysis. Peer J.

[14] Haider BA, Olofin I, Wang M, Spiegelman D, Ezzati M, Fawzi WW (2013). Anaemia, prenatal iron use, and risk of adverse pregnancy outcomes: Systematic review and meta-analysis. Br Med J.

[15] Kelly Y, Panico L, Bartley M, Marmot M, Nazroo J, Sacker A (2009). Why does birthweight vary among ethnic groups in the UK? Findings from the millennium cohort study. J Public Health.

[16] Villar J, Papageorghiou AT, Pang R, Ohuma EO, Ismail LC, Barros FC (2014). The likeness of fetal growth and newborn size across non-isolated populations in the INTERGROWTH-21 st project: the fetal growth longitudinal study and newborn cross-sectional study. Lancet Diabetes Endocrinol.

[17] Papageorghiou AT, Kennedy SH, Salomon LJ, Altman DG, Ohuma EO, Stones W, Gravett MG, Barros FC, Victora C, Purwar M, Jaffer Y, Noble JA, Bertino E, Pang R, Cheikh Ismail L, Lambert A, Bhutta ZA, Villar J (2018). The INTERGROWTH-21st fetal growth standards: toward the global integration of pregnancy and pediatric care. Am J Obstet Gynecol.

[18] Kozuki N, Lee ACC, Silveira MF, Sania A, Vogel JP, Adair LS, Barros F, Caulfield LE, Christian P, Fawzi W, Humphrey J, Huybregts L, Mongkolchati A, Ntozini R, Osrin D, Roberfroid D, Tielsch J, Vaidya A, Black RE, Katz J, Child Health Epidemiology Reference Group (CHERG) Small-for-Gestational-Age-Preterm Birth Working Group (2013). The associations of parity and maternal age with small-for-gestational-age, preterm, and neonatal and infant mortality: a meta-analysis. BioMed Central Public Health.

[19] Shapiro GD, Bushnik T, Wilkins R, Kramer MS, Kaufman JS, Sheppard AJ (2018). Adverse birth outcomes in relation to maternal marital and cohabitation status in Canada. Ann Epidemiol.

[20] Shah PS, Zao J, Ali S (2011). Maternal marital status and birth outcomes: a systematic review and meta-analyses. Matern Child Health J.

[21] Ciobanu A, Rouvali A, Syngelaki A, Akolekar R, Nicolaides KH (2019). Prediction of small for gestational age neonates: Screening by maternal factors, fetal biometry, and biomarkers at 35–37 weeks’ gestation. Am J Obstetr Gynecol.

[22] Blackburn S (2013). Maternal, fetal and neonatal physiology.

[23] Rafei RE, Abbas HA, Alameddine H, Bizri AA, Melki I, Yunis KA (2018). Assessing the risk of having small for gestational age newborns among Lebanese underweight and normal pre-pregnancy weight women. Matern Child Health J.

[24] Pugh SJ, Albert PS, Kim S, Grobman W, Hinkle SN, Newman RB (2017). Patterns of gestational weight gain and birthweight outcomes in the Eunice Kennedy Shriver National Institute of Child Health and Human Development fetal growth studies–singletons: a prospective study. Am J Obstetr Gynecol.

[25] Anderson NH, Sadler LC, Stewart AW, Fyfe EM, McCowan LME (2013). Independent risk factors for infants who are small for gestational age by customised birthweight centiles in a multi-ethnic New Zealand population. Aust N Z J Obstet Gynaecol.

[26] Goldstein RF, Abell SK, Ranasinha S, Misso M, Boyle JA, Black MH, Li N, Hu G, Corrado F, Rode L, Kim YJ, Haugen M, Song WO, Kim MH, Bogaerts A, Devlieger R, Chung JH, Teede HJ (2017). Association of gestational weight gain with maternal and infant outcomes: a systematic review and meta-analysis. J Am Med Assoc.

[27] Pollard CM, Booth S (2019). Food insecurity and hunger in rich countries-it is time for action against inequality. Int J Environ Res Public Health.

[28] Morisaki N, Urayama KY, Yoshii K, Subramanian SV, Yokoya S (2017). Ecological analysis of secular trends in low birth weight births and adult height in Japan. J Epidemiol Community Health.

[29] Getnet W, Aycheh W, Tessema T (2018). Determinants of food taboos in the pregnant women of the Awabel District, East Gojjam zone, Amhara Regional State in Ethiopia. Adv in Public Health.

[30] Zerfu TA, Umeta M, Baye K (2016). Dietary habits, food taboos, and perceptions towards weight gain during pregnancy in Arsi, rural central Ethiopia: A qualitative cross-sectional study. J Health Popul Nutr.

[31] Bushnik T, Yang S, Kaufman JS, Kramer MS, Wilkins R (2017). Socioeconomic disparities in small-for-gestational-age birth and preterm birth. Health Rep.

[32] Hirst JE, Knight HE, Ohuma EO, Dwyer T, Hennig BD, Papageorghiou AT (2018). Social gradient of birthweight in England assessed using the INTERGROWTH-21st gestational age-specific standard. Arch Dis Child Fetal Neonatal Ed.

[33] Hendrix NMD, Berghella VMD (2008). Non-placental causes of intrauterine growth restriction. Semin Perinatol.

[34] Kayode GA, Amoakoh-Coleman M, Akua Agyepong I, Ansah E, Grobbee DE, Klipstein-Grobusch K (2014). Contextual risk factors for low birth weight: A multilevel analysis. Plos One.

[35] Cetin I, Mandò C, Calabrese S (2013). Maternal predictors of intrauterine growth restriction. Curr Opin Clin Nutr Metab Care.

[36] Coleman S (2016). Built environment: Increased extreme weather events. Australia state of the environment 2016.

[37] David M, Borde T, Brenne S, Ramsauer B, Henrich W, Breckenkamp J, Razum O (2017). Obstetric and perinatal outcomes among immigrant and non-immigrant women in Berlin. German Arch Gynecol Obstetr.

[38] Slaughter-Acey JC, Holzman C, Calloway D, Tian Y (2016). Movin’ on up: socioeconomic mobility and the risk of delivering a small-for-gestational age infant. Matern Child Health J.

[39] Kothari CL, Paul R, Dormitorio B, Ospina F, James A, Lenz D (2016). The interplay of race, socioeconomic status and neighborhood residence upon birth outcomes in a high black infant mortality community. Soc Sci Med J Popul Health.

[40] Eskes M, Abu-Hanna A, Ravelli ACJ, Waelput AJM, Scherjon SA, Bergman KA (2017). Small for gestational age and perinatal mortality at term: an audit in a Dutch national cohort study. Eur J Obstetr Gynecol Reprod Biol.

[41] Slaughter-Acey JC, Talley LM, Stevenson HC, Misra DP (2019). Personal versus group experiences of racism and risk of delivering a small-for-gestational age infant in African American women: a life course perspective. J Urban Health.

[42] Heslehurst N, Brown H, Pemu A, Coleman H, Rankin J (2018). Perinatal health outcomes and care among asylum seekers and refugees: a systematic review of systematic reviews. BioMed Central Med.

[43] Migration: Australia. ABS. 2020. Available from: https://www.abs.gov.au/statistics/people/population/migration-australia/latest-release#net-overseas-migration. [cited 20/12/2020].

[44] Australia's mothers and babies data visualisations. AIHW. 2020. Available from: https://www.aihw.gov.au/reports/mothers-babies/australias-mothers-babies-2017-data-visualisations/contents/demographics-of-mothers-and-their-babies/maternal-country-of-birth. [cited 18/12/2020]

[45] Births, Australia, 2017. ABS website. 2018. Available from: https://www.abs.gov.au/AUSSTATS/abs@.nsf/Latestproducts/3301.0Main%20Features32017?opendocument&tabname=Summary&prodno=3301.0&issue=2017&num=&view=. [cited 20/12/2020]

[46] Davies-Tuck ML, Davey M-A, Wallace EM (2017). Maternal region of birth and stillbirth in Victoria, Australia 2000–2011: A retrospective cohort study of Victorian perinatal data. Plos One.

[47] Belihu FB, Davey M-A, Small R (2016). Perinatal health outcomes of East African immigrant populations in Victoria, Australia: A population based study. BioMed Central Pregnancy and Childbirth.

[48] Davey M-A, Sloan M-L, Palma S, Riley M, King J (2013). Methodological processes in validating and analysing the quality of population-based data: a case study using the Victorian perinatal data collection. Health Information Manage J.

[49] Lausman A, Kingdom J (2013). Intrauterine growth restriction: screening, diagnosis, and management. J Obstet Gynaecol Can.

[50] Dobbins TA, Sullivan EA, Roberts CL, Simpson JM (2012). Australian national birthweight percentiles by sex and gestational age, 1998–2007. Med J Aust.

[51] Selvaratnam RJ, Davey M-A, Wallace EM (2020). The pitfalls of using birthweight centile charts to audit care. Plos One.

[52] Gardosi J, Francis A, Turner S, Williams M (2018). Customized growth charts: rationale, validation and clinical benefits. Am J Obstet Gynecol.

[53] Standard Country or Area Codes For Statistical Use (M49). 2011. Available from: https://unstats.un.org/unsd/methodology/m49/. [cited 11/07/2020]

[54] Victorian Government (2019). Victorian perinatal data collection manual, version 7.0.

[55] Australian Bureau of Statistics (2016). 1269.0-Standard Australian Classification of Countries (SACC).

[56] Australian Bureau of Statistics. Technical paper: socio-economic indexes for areas (SEIFA) 2016. ACT: Commonwealth of Australia; 2018.

[57] Lowe SA, Bowyer L, Lust K, McMahon LP, Morton MR, North RA (2014). Guideline for the Management of Hypertensive Disorders of Pregancy.

[58] Position statement: detection and management of fetal growth restriction in singleton pregnancies. Sydney: Centre of Research Excellence in Stillbirth. 2019.

[59] United Nations. International migration 2019: highlights. South Brisbane: Department of Economic and Social Affairs PD; 2019.

[60] Sustainable Development Goals. United Nations. 2019. Available from: https://www.un.org/sustainabledevelopment/. Accessed 1 Dec 2020.

[61] Australian Human Rights Commision (2016). Immigration detention and human rights.

[62] Villalonga-Olives E, Kawachi I, von Steinbüchel N (2016). Pregnancy and birth outcomes among immigrant women in the US and Europe: a systematic review. J Immigr Minor Health.

[63] Pangas J, Ogunsiji O, Elmir R, Raman S, Liamputtong P, Burns E, Dahlen HG, Schmied V (2019). Refugee women's experiences negotiating motherhood and maternity care in a new country: a meta-ethnographic review. Int J Nurs Stud.

[64] Boucher A (2019). Measuring migrant worker rights violations in practice: the example of temporary skilled visas in Australia. J Ind Relat.

[65] AWE K, Egan M, Tabbner C, Tannahill C (2017). Healthy migrants in an unhealthy city? The effects of time on the health of migrants living in deprived areas of Glasgow. J Int Migr Integr.

[66] Eskild A, Sommerfelt S, Skau I, Grytten J. Offspring birthweight and placental weight in immigrant women from conflict-zone countries; does length of residence in the host country matter? A population study in Norway. Acta Obstet Gynecol Scand. 2019;99(5):615-22. 10.1111/aogs.13777. 10.1111/aogs.13777 31774545

[67] Sørbye IK, Vangen S, Juarez SP, Bolumar F, Morisaki N, Gissler M, et al. Birthweight of babies born to migrant mothers - what role do integration policies play? Soc Sci Med. 2019;9:1–7. 10.1016/j.ssmph.2019.100503. 10.1016/j.ssmph.2019.100503 PMC6978482 31993489

[68] Reiss K, Breckenkamp J, Borde T, Brenne S, David M, Razum O (2015). Contribution of overweight and obesity to adverse pregnancy outcomes among immigrant and non-immigrant women in Berlin. German Eur J Public Health.

[69] Klapdor M (2020). Temporary visa holders and social security: A quick guide: Parlament of Australia.

[70] Australian Government (2020). Immigration and citizenship.

[71] Gibson-Helm M, Boyle J, Cheng IH, East C, Knight M, Teede H (2015). Maternal health and pregnancy outcomes among women of refugee background from Asian countries. Int J Gynecol Obstet.

[72] Puthussery SMSW (2015). Perinatal outcomes among migrant mothers in the United Kingdom: is it a matter of biology, behaviour, policy, social determinants or access to health care?. Best Pract Re.

[73] Yelland J, Riggs E, Wahidi S, Fouladi F, Casey S, Szwarc J (2014). How do Australian maternity and early childhood health services identify and respond to the settlement experience and social context of refugee background families?. BioMed Central Pregnancy Childbirth.

[74] Fox H, Topp SM, Callander E, Lindsay D (2019). A review of the impact of financing mechanisms on maternal health care in Australia. BMC Public Health.

[75] Yelland J, Riggs E, Small R, Brown S (2015). Maternity services are not meeting the needs of immigrant women of non-English speaking background: results of two consecutive Australian population based studies. Midwifery..

[76] Mander S, Miller YD (2016). Perceived safety, quality and cultural competency of maternity care for culturally and linguistically diverse women in Queensland. J Racial Ethn Health Disparities.

[77] Yelland J, Mensah F, Riggs E, McDonald E, Szwarc J, Dawson W, Vanpraag D, Casey S, East C, Biro MA, Teale G, Willey S, Brown SJ (2020). Evaluation of systems reform in public hospitals, Victoria, Australia, to improve access to antenatal care for women of refugee background: an interrupted time series design. Plos Med.

[78] Yelland J, Riggs E, Szwarc J, Casey S, Dawson W, Vanpraag D (2015). Bridging the Gap: Using an interrupted time series design to evaluate systems reform addressing refugee maternal and child health inequalities. Implement Sci.

[79] MacDonald TM, McCarthy EA, Walker SP (2015). Shining light in dark corners: diagnosis and management of late-onset fetal growth restriction. Aust N Z J Obstet Gynaecol.

[80] Henrichs J, Verfaille V, Jellema P, Viester L, Pajkrt E, Wilschut J, et al. Effectiveness of routine third trimester ultrasonography to reduce adverse perinatal outcomes in low risk pregnancy (the IRIS study): nationwide, pragmatic, multicentre, stepped wedge cluster randomised trial. Br Med J. 2019:l5517. 10.1136/bmj.l5517. 10.1136/bmj.l5517 PMC6792062 31615781

